# Assessing Pancreas Transplant Candidate Cardiac Disease: Preoperative Protocol Development at a Rapidly Growing Transplant Program

**DOI:** 10.3390/mps2040082

**Published:** 2019-10-17

**Authors:** David St. Michel, Tracy Donnelly, Towanda Jackson, Bradley Taylor, Rolf N. Barth, Jonathan S. Bromberg, Joseph R. Scalea

**Affiliations:** Department of Surgery, University of Maryland, Baltimore, MD 20742, USA; dmichel@som.umaryland.edu (D.S.M.); tracy.donnelly@umm.edu (T.D.); towanda.jackson@umm.edu (T.J.); btaylor@som.umaryland.edu (B.T.); rbarth@som.umaryland.edu (R.N.B.); jbromberg@som.umaryland.edu (J.S.B.)

**Keywords:** transplant, pancreas, cardiac, preoperative

## Abstract

Pancreas transplant rates, despite improving outcomes, have decreased over the past two decades. This is due, in part, to ageing, increasingly co-morbid pancreas transplant candidates. There is a paucity of published data regarding coronary artery disease (CAD) in this population. To inform peri-operative management strategies, we sought to understand the frequency of CAD among recipients of pancreas transplants at our center. Informed by these data, we sought to develop a standard protocol for evaluation. A retrospective review of pancreas transplants (solitary pancreas and simultaneous pancreas-kidney) was undertaken at the University of Maryland. Transplant outcomes and frequency of cardiac disease were analyzed. Current data were compared with historic controls. Over the study period, 59 patients underwent pancreas transplantation. Coronary architecture was assessed in 38 patients (64.4%). Discrete evidence of CAD was present in 28 of 39 patients (71.7%). All pancreas candidates (n = 21) who underwent left heart catheterization (LHC) demonstrated CAD (100%). No patients experienced myocardial infarction (MI) and no deaths resulted from cardiac disease in the early post-transplant period. Pancreas transplant candidates are at high risk for CAD. At a center in which pancreas transplant rates are increasing, a rigorous cardiac work up revealed that 71.7% of assessed recipients had CAD. Although asymptomatic, 6.8% required coronary artery bypass graft (CABG). Despite increasing age and co-morbid status, pancreas transplant recipients can enjoy excellent results if protocolized preoperative testing is used.

## 1. Introduction

Pancreas transplant rates have fallen from a peak of nearly 1500 in 2004 to fewer than 1000 transplants in 2018 [1,2]. Indeed, simultaneous pancreas and kidney (SPK) transplantation rates fell 10% annually from 2004–2011, while decreases in pancreas transplant alone (PTA) and pancreas after kidney (PAK) have been more dramatic (34% and 55%, respectively) [1,2,3]. As of 2016, only 11 centers nationally performed more than 20 pancreas transplants annually [1]. Reasons for falling transplant rates are not completely understood, but are thought to include improved diabetes management, reduced training opportunities, increased scrutiny of outcomes, and a decreasing number of surgical pancreas transplant referrals [4].

Pancreas transplant outcomes have continued to improve over the last 3 decades. Improved patient and graft survivals are likely the result of improved surgical technique, preoperative screening, and postoperative management [4]. For SPKs, pancreas graft survivals at 1 and 3 years have increased in the last decade from 95.2% and 90.9%, to 97.2% and 94.3%, respectively [1,5]. Further, pancreas transplantation improves quality of life, and extends life expectancy beyond kidney transplantation alone [6,7,8]. 

The medical complexity of modern-era pancreas transplant recipients is uniquely challenging [9,10]. Over the past decade several trends have been noted among candidates: a higher proportion are greater than 50 years of age, obesity has increased, and more type II diabetic candidates are listed [4,5]. With the changing profile of pancreas transplant candidates, there is a building need for surgical literature which addresses how these risk factors affect surgical outcomes. 

The incidence of coronary artery disease (CAD) in the pancreas transplant population is surprisingly understudied. Diabetes is a leading risk factor for CAD. To this end, patients undergoing SPK and simultaneous pancreas (SP) transplantation are at higher risk for cardiac ischemia following surgery [11,12] In pancreas transplant recipients, the risk of perioperative cardiovascular events may exceed 10% [13] and perioperative MI associated with a mortality of 3–25% [14]. Indeed, cardiovascular disease is the most common cause of death after SPK and death is the most common cause of graft loss after SPK [15]. 

Starting in 2016, our center observed a large increase in pancreas transplant volumes [16]. Our center has also noted a trend towards older, heavier transplant recipients [16]. Recognizing the elevated, yet unknown risk for CAD in this changing patient population, we have used a rigorous approach to cardiac testing in the pre-transplant setting [10]. Here we sought to determine, among transplanted patients, the frequency of CAD and to determine if our pre-operative testing was supported by good outcomes after transplantation. We hypothesized that a preponderance of patients undergoing pancreas transplantation had CAD. In retrospective analysis, we found that CAD affected more than two-thirds of those who underwent coronary artery assessment (CAA). The findings from the present study have informed our institution’s cardiac pre-operative testing as well as our approach to patient selection. These findings suggest that with a rigorous cardiac assessment, pancreas transplant recipients can enjoy excellent post-transplant outcomes.

## 2. Methods

**Patients:** Upon Internal Review Board (IRB) approval, a retrospective study of adult-only transplant recipients at the University of Maryland Medical Center was undertaken. All patients evaluated for (and transplanted with) a pancreas over the study period were included. Our data included patients undergoing pancreas after kidney (PAK), pancreas transplant alone (PTA), and simultaneous pancreas-kidney (SPK) transplantation. All consecutive transplants were included. 

**Patient acceptance:** Patients were evaluated in multi-disciplinary transplant clinic. As has been published, high volumes of pancreas transplant candidates have been observed in our institution through a comprehensive educational system which allows schedulers, medical assistants, nursing coordinators, surgeons, and medical practitioners to identify pancreas and kidney-pancreas transplant patients in the outpatient setting [16]. Patients are presented by nursing coordinators at weekly multi-disciplinary transplant listing meetings. If accepted, a dedicated pancreas transplant nurse coordinator and surgical director co-manage all pancreas transplant work ups.

**Cardiac testing:** Cardiac function and coronary work ups were suggested by a multi-disciplinary team of surgeons, cardiologists, and nephrologists. In general, pancreas transplant candidates with >10 years of diabetes, a prior CVA, a prior MI, or those with concerning findings on either echocardiogram or stress tests were referred for coronary architecture assessment. The preference of the transplant team is to obtain CT imaging with calcium scoring, in advance of invasive angiography, in order to limit potential procedural morbidity. In some cases, these cardiac assessments were done in outside hospital settings where practitioners elected for cardiac catheterization without CT. However, when patients were evaluated at the author’s center, cardiac CT scan with calcium scoring was obtained in advance of LHC. CT scans were assessed using a protocol built for cardiology, cardiac surgery, and transplant surgery. In general, when the calcium score was low (<160 by the Agatston method), no additional work up was required if echocardiography and stress testing were also unconcerning. If the calcium score was higher, patients were generally sent for LHC. All cardiac work ups were completed and verified in a second cardiac work up meeting prior to listing. 

**Coronary artery disease definition:** CAD was defined as the presence of coronary atherosclerosis on CT scan (with or without contrast), coronary angiogram (LHC), or a history of prior and documented MI or CABG. 

**Comparison group:** There was no internal comparison group for cardiac workup amongst the patients in this study. As such, a group of patients from 2013, 2014, and 2015 (n = 50) were included as a historic comparator group. Demographics, laboratory values, and transplant surgical data were collected. 

**Graft failures:** Kidney failure was defined as return dialysis after freedom from dialysis, primary non-function of the organ, or death with a functional graft. Pancreas failure was defined as return to insulin, primary non-function (never came off insulin), pancreatectomy, or death with functional graft. 

**Endpoints:** Data was analyzed to determine the prevalence of CAD in our study population. Data on patient pancreas graft survival and troponin elevation within 30 days of transplantation were also analyzed.

**Statistical analysis:** Statistical analysis was performed using the T-test, Fischer’s exact test, Chi-square and Log-rank analyses where appropriate. The statistical package used was SPSS. Historical data were compared with current data where relevant. 

## 3. Results

### 3.1. Pancreas Demographics, Transplants, and Outcomes

From 1 January 2017 to 14 May 2018, a total of 59 pancreas transplants were performed (Table 1). Among these, five (8.5%) were PTA, eight (13.5%) were PAK, and 46 were SPKs (78.0%). In addition, six were second pancreas transplants and two were third pancreas transplants. The mean and median recipient ages were 46.8 and 47.0 years, respectively (range: 29–66). Twenty-two patients (37.2%) were aged 50 or older. Thirty-five patients (59.3%) were Caucasian, 20 (33.8%) were African American, three (5.1%) were Hispanic and one (1.7%) was Asian. Thirty-four (57.6%) recipients were on hemodialysis (HD) prior to transplantation.

Donors had a mean and median KDPI of 29% and 27%, respectively. The mean and median cold ischemia times for pancreas transplants were 10.1 h and 7.9 h, respectively. Twenty-six (44.1%) of donors were considered to have an increased risk of contracting PHS. Of the 26 PHS increased risk donors, three (11.5%) were transplanted as PTAs, four (15.4%) as PAK, and 19 (73.1%) as SPKs.

**Figure 1:**
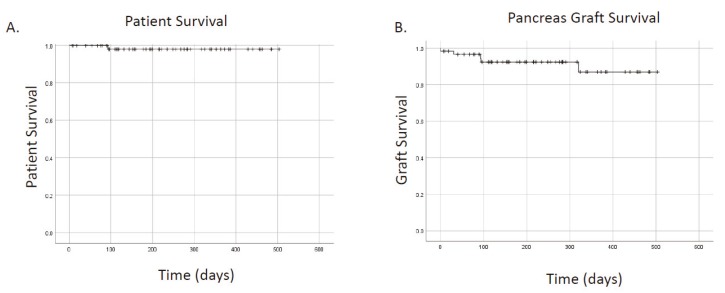
Patient and graft survival for recipients of pancreas transplants. (**A**) patient survival. (**B**) graft survival.

**Figure 2:**
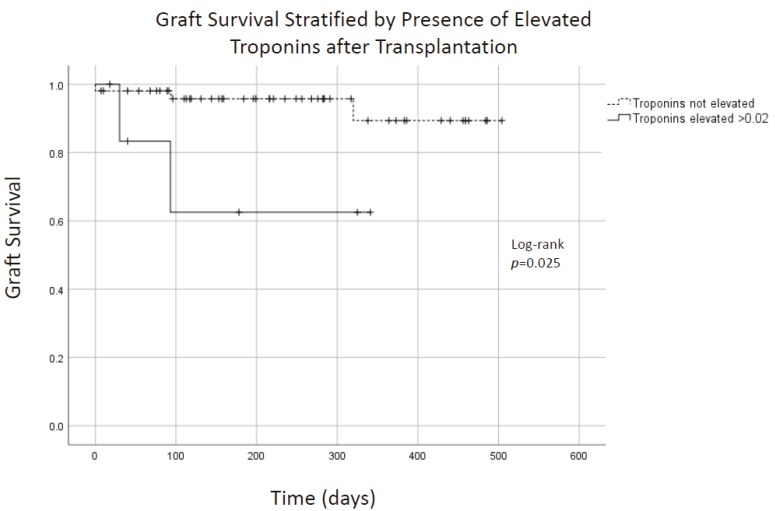
Graft survival and troponin elevation after pancreas transplantation. Graft survival was worse for patients who also experienced post-transplantation troponin elevation (*p* = 0.025).

Mean and median follow up were 7.1 and 6.6 months, respectively. No patients were lost to follow up. A single patient (1.7%) expired. In total, five grafts failed. Causes of graft failure are presented in Table 2. Two grafts (3.4%) failed from rejection (one in a highly sensitized patient and the second from non-compliance). One graft failed from thrombosis (1.7%). Overall patient survival was 98.3%, overall kidney survival was 95.6%, and overall pancreas survival was 91.5% (Figure 1A,B). Thirty-day pancreas graft survival was 96.5%. With short follow up, pancreas graft survivals were not different for primary versus re-transplanted patients (*p* = 0.72). Graft survival appeared to be lower in patients who had troponin elevation within 30 days of transplantation (Figure 2; *p* = 0.025). The mean creatinine for SPK recipients at follow-up was 1.24 mg/dL (Table 3). There was no difference in follow-up creatinine between SPK and PTA recipients (*p* = 0.23) or PAK and PTA recipients (*p* = 0.12). Death censored renal graft survival was 100%.

### 3.2. Echocardiograms and Stress-Testing among Transplanted Patients

All patients (n = 59) had an echocardiogram (echo) and a stress test prior to transplantation. The mean and median ejection fraction (EF) reported were 61.2% and 60.0%, respectively (range: 25–85%). Nine patients (15.3%) had a dobutamine stress test (DST), 14 patients (23.7%) had an exercise stress test (EST), and 38 patients (59.3%) had Sestimibi myocardial perfusion rest-stress tests using single photon emission computed tomography (SPECT). Two patients (3.4%) had both EST and SPECT, and one patient (1.7%) had both DST + SPECT. A single patient (1.7%) had a cardiac PET scan alone. Out of all the stress tests, three (5.1%) were reported as positive, two (3.4%) as indeterminate, and the remainder were negative. Positive stress tests uniformly led to assessment of coronary architecture with LHC or CTA. 

### 3.3. Coronary Artery Architecture Assessment

Overall, 28 of 59 transplanted patients (47.5%) had discrete evidence of CAD. However, when we considered only those who underwent discrete evaluation of coronary architecture, we identified that 28 of 39 patients (71.7%) had CAD. 

**CT imaging:** 38 patients (64.4%) out of 59 had coronary architecture assessment prior to transplantation. Detailed calcium score data (n = 24), where available, is presented in Table 4. An additional patient had a known history of CAD, and underwent CABG, yielding a total of 39 patients with known CAD. Twenty seven of 59 patients (45.8%) underwent CT imaging of the coronary arteries, and an additional 11 (18.6%) underwent LHC, without prior CT. Based on cardiac CT results, 10 patients of 27 were sent for subsequent LHC. A total of 21 patients in our population had an LHC performed. 

**Left heart catheterization:** 21 patients (35.5%) underwent left-sided cardiac catheterization (Table 5). A single LHC was performed for a patient with a calcium score of <160 because cardiac CT angiogram performed for this patient showed an isolated stenosis of 75% in the mid-LAD. All 21 patients had identifiable coronary disease on LHC. 

**Cardiac stenting:** Four patients underwent cardiac stenting before pancreas transplant testing. Three of these were to the left coronary and one was to the right coronary. Two of these patients had in-stent stenosis on cardiac catheterization during pre-pancreas transplant testing. No transplanted patients were stented as a direct result of pancreas transplant work up. 

**Coronary artery bypass grafting:** Four patients (6.8%) underwent coronary artery bypass grafting (CABG). The results of pancreas transplant testing LHCs led to CABG for 3 transplanted patients (5.1%). A single, additional patient underwent previously scheduled CABG during his pancreas transplant work up.

### 3.4. Cardiac Outcomes

Mean and median follow up were 7.1 and 6.6 months, respectively. No patients were lost to follow up. A single patient (1.7%) expired. No patients had post-transplantation MI. Seventeen patients (28.8%) had a troponin checked within 30 days of pancreas transplantation. Of these, seven (41.1%) were elevated above 0.02, which is the institution’s upper limit of normal for troponin. The highest troponin was 8.51, and this occurred after respiratory arrest in our sole patient death. Excluding this patient, the highest troponin was 1.93. All remaining (n = 5) elevated troponins were ≤0.81. Eight of the 17 patients (47.1%) for whom troponins were checked had undergone a LHC prior to transplantation. Two patients of the 11 who underwent CT followed by LHC had an elevated troponin (>0.02) after transplantation, while zero patients of the 17 who underwent CT imaging alone (*p* = 0.38) demonstrated elevation (Table 6).

### 3.5. Construction of Formal Cardiac Protocol for Pancreas Evaluation

**Figure 3:**
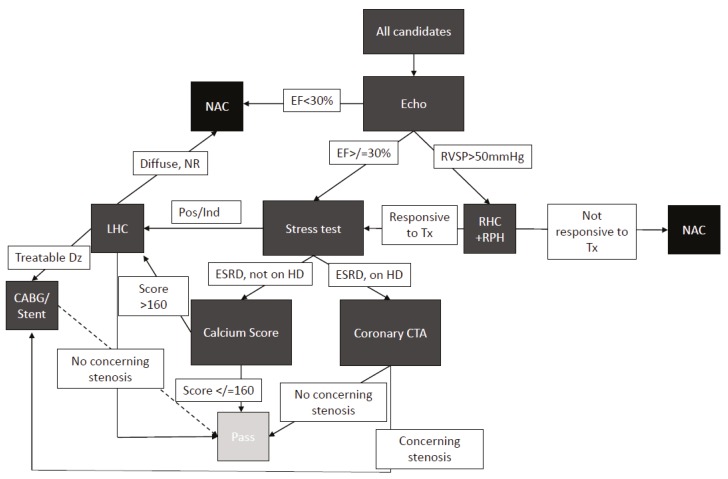
Protocol for cardiac work up in pancreas transplant candidates.

Informed by the above data we developed a protocolized approach to pancreas transplant candidate evaluation (Figure 3). All patients received electrocardiogram (ECG), chest radiograph (CXR), and echocardiogram. If the echocardiogram shows EF < 30%, then the patient is not a candidate. If the echocardiogram shows elevated right-ventricular diastolic pressure, then patients are referred for a right-heart catheterization and pulmonary medicine recommendations. If the EF is ≥30%, patients undergo coronary assessment with either calcium scoring or with a CT of the coronary arteries, which may prompt a left-heart catheterization. If the left-heart catheterization shows a disease requiring treatment, the patients undergo either stenting or CABG. Following CABG or stent, patients undergo repeat stress test after six weeks, and if a negative result is obtained, they continue through the listing process. 

### 3.6. Comparison with Center-Specific Historical Data

In the years 2013, 2014, and 2015, a total of 50 consecutive pancreas transplants were performed. Of these 29 were SPK (58%), 11 were PTA (22%), and 10 were PAK (20%). The mean and median age were 45.4 and 46 years, and the mean and median BMI were 25 and 24.25. Thirty four patients (68%) were Caucasian, 12 were African American (24%), and four were classified as other (8%). In comparison to 17 out of the 59 (34%) patients in the current group, in the historical group 10 of the 50 (20%) patients had troponins checked within 30 days of transplantation (*p* < 0.29). When compared with the most recent data, the historical patients had no increased rate of troponin elevation in those for whom troponins were checked. Five of the 50 historic patients (10%) underwent an LHC prior to transplantation, which was significantly lower than the current cohort (*p* < 0.01). 

## 4. Discussion

Coronary disease is a significant concern before and after pancreas transplantation [10,17,18]. Perhaps resulting from decreased surgical volumes, the true frequency of CAD amongst recipients of pancreas transplants is understudied. Knowledge of the burden of CAD in the modern era is particularly important as patients are getting older before they are referred for transplantation [5,10]. Recognizing increasing volumes at our center in the setting of an aging and increasingly co-morbid pancreas transplant population, we sought to determine how many of our pancreas transplant recipients were affected by CAD [16]. Our data showed that more than two-thirds of pancreas transplant candidates who underwent CAA pre-transplantation had CAD. 

A study utilizing the National Inpatient Sample data demonstrated that preoperative pancreas transplant patients (SPK, PAK and PTA) had a 69.8% incidence of HTN, 3.3% had a prior coronary bypass, 4.8% had a prior coronary intervention, and 1.9% had known valvular disease, indicating a likely high prevalence of cardiac in this population [19]. The SPK population is likely at higher risk of CAD when compared to pancreas transplantation alone. Patients with end-stage renal disease, in the presence of diabetes and other comorbidities, are at a 10-fold higher risk of developing cardiovascular complications [15,19]. These patients are known to have high rates of HTN (79.5%), peripheral vascular disease (4.9%), and prior coronary intervention (4%) [19]. A vigilant approach is required in the pre-transplant setting [11]. 

Patients in the current study were more likely to undergo cardiac catheterization when compared with historic controls. This may have helped to avoid cardiac events after transplantation. In our experience, every pancreas transplant (100%) who underwent LHC had identifiable coronary disease of varying degrees. This universal presence of CAD is higher than previously reported. Data presented by our institution more than a decade ago showed that out of patients who underwent pre-pancreas transplant catheterization, 54% had significant coronary artery stenoses [18]. It is likely that our observed rates of CAD were higher than previously reported because, in many cases, our patients were pre-screened with cardiac CT scans before catheterization. However, it is possible that we are seeing an older, sicker population presenting for transplantation. A formal comparison of our center’s historic and present groups was not the focus of the present investigation. Table 5 provides data for the 21 patients in this study who underwent LHC. Each demonstrates CAD. 

The revised cardiac risk index (RCRI, or revised Lee risk index), the most commonly used preoperative risk score, assigns 1 point for each of: high-risk surgery, ischemic heart disease, congestive heart failure, cerebrovascular disease, insulin-dependent diabetes mellitus, and renal failure (creatinine >2.0 mg/dL) [11]. Patients with 3 more than points are considered high risk [11,20]. With a RCRI index score of ≥3, surgery carries an 11% risk of major cardiac event [11]. Based on these criteria, nearly all patients undergoing SPK and SP transplantation fall into the highest cardiac risk category. 

Despite their asymptomatic status, three cases of CAD in this series was severe enough to warrant CABG (an additional patient underwent a previously scheduled CABG during preoperative evaluation). This may reflect the reduced symptoms of CAD and ischemia among diabetic patients [21,22]. Perhaps notably, cardiac stenting was not utilized during our pre-pancreas transplant work ups. We have reasoned that this was based on surgeon preference for patients to be off clopidogrel and other non-aspirin antiplatelet agents (which are often required after cardiac stenting) at the time of transplantation. However, since data was collected for this manuscript, newer stents that require shorter clopidogrel durations (two weeks) have become available and these stents have since been used in our center’s pancreas transplant population. 

One interpretation of these data is that the lack of stenting amongst transplanted candidates argues against such rigid CAA in advance of pancreas transplantation. That is, why perform these LHC’s if no one has a stent? On the other hand, three patients required CABG as a direct result of the CAA required for pancreas transplantation. Further, this paper does not address the patients who did not qualify for pancreas transplantation because of the pancreas transplant cardiac evaluation. Indeed, three additional patients (data not shown) evaluated during the time frame in which this study’s patients were transplanted, underwent CABG before pancreas listing. One of these patients expired prior to receiving an offer and the other two were on the waiting list. Because not all patients are deemed transplant candidates after cardiac work up, there is a selection bias to explain (in part) our better-than-expected cardiac outcomes. Additional studies could be designed to determine how many patients were declined transplant as a result of cardiac testing and to better understand the appropriateness of CAA modality (i.e., CT vs. LHC vs. no-testing). The goal of the present study was to determine, among transplanted patients, the frequency of CAD and to determine if our pre-operative testing was supported by good outcomes after transplantation. We found that CAD was quite common and that it should be carefully considered in advance of pancreas transplant surgery. 

In a study designed to determine if cardiac CT and calcium scoring added value to the preoperative work up, authors found that higher Revised Cardiac Risk Index (RCRI) plus high calcium score (>113) were together more predictive of post-operative cardiac events than RCRI alone [12]. At our institution, we have adopted a calcium score “cutoff” of 160 [12,23,24]. This was chosen because a calcium score of >160 is associated with a higher rate of non-fatal and fatal MI when compared with those who have a score <160 [23]. We observed a low incidence of troponin elevation and a general avoidance of cardiac events. 

Observed elevations in troponins were interpreted as myocardial strain, and none were thought by cardiology consultants to be clinically significant MI. Why troponin elevation was correlated with graft failure is not clear, but suggests that patients with at-risk myocardium may be at higher risk for graft failure. Renal function may have also contributed to observed elevations because troponins are cleared renally. These data may suggest that clinically significant myocardial infarctions early in the post-operative period can be avoided using a robust pre-transplant assessment paired with careful patient selection. 

There are several shortcomings in this study. Firstly, there was no internal comparison group, so historic data were gathered for comparison. In doing so, we observed no difference in rate of troponin elevation or the frequency with which troponins were checked after transplantation when the current cohort was compared with historic data. This should be qualified by the knowledge that there was no preexisting protocol for cardiac assessment. While there was no difference in age or BMI between the historic and the current groups, our group’s transplant volume was much higher (59 in two years vs. 50 in three years) when compared with historic data. Despite this, there was no increase in the rate of major cardiac events. To this end, our current protocol may support rapid growth of a pancreas transplant program with no observed increased risk of cardiac complication. Another shortcoming is the small size of this study. For example, it is plausible that we have encountered a type II statistical error and that we simply have not yet transplanted enough patients to determine if there is a true difference in cardiac risk with or without or protocol. Larger studies, perhaps multi-center, could help offset this risk in the future. However, given the severity of illness in this patient population, it occurred to the authors that an added level of caution with regard to the cardiac work up for patients undergoing pancreas transplantation may be warranted. 

Cardiac disease is not synonymous with coronary disease. The mean and median EFs observed in this study were in the normal range. To this end, it may be important to highlight that heart function (rather than perfusion) among our diabetic renal failure patients was preserved. This finding suggests that although a pancreas transplant candidate may have reasonable functional status, their myocardium may still be at risk [21,22]. Further, the observation that our transplanted patients had good cardiac function again speaks to the importance of patient selection. 

As a result of this analysis, we have implemented a standard protocol for cardiac evaluation in the pancreas transplant population. This protocol is imperfect. However, it allows our staff the flexibility and ease of moving patients efficiently through a complex system. Our protocol also offers an opportunity to study cardiac outcomes in a standardized fashion. Our retrospective results suggest that we are avoiding significant coronary disease in the pancreas transplant population. Given the small number of patients in this study, future prospective studies could address this protocol’s safety and efficacy.

A potential benefit of the proposed protocol is to improve the objective nature of the transplant evaluation process. Indeed, a protocolized system simplifies decision making in a rapidly growing system. In the historic data set, fewer patients were transplanted per time, allowing for more individualized decision making. This is a less efficient process in a rapidly growing system. Differences in efficiency (e.g., days from evaluation to cardiac clearance, days from transplant evaluation to cardiac evaluation, days from evaluation to listing, numbers of canceled appointments, total healthcare dollars spent, etc.) were not collected and were thus not available for comparison. However, future studies investigating the benefits of cardiac protocolization could focus on determining which elements of the proposed system are more efficient than alternatives. 

Pancreas transplantation in the modern era provides rapid access to high-quality organs which render recipients euglycemic and, for SPK, independent of dialysis. Pancreas transplant candidates may be sicker than they appear, and more than two-thirds have coronary artery disease, despite preserved EF. While there was no discrete decrease in retrospectively identified cardiac events, implementation of our cardiac protocol allowed our pancreas transplant program to expand rapidly without additional cardiac events. To this end, our center has employed a rigorous and protocolized approach to minimize risk for patients with at-risk myocardium.

## Figures and Tables

**Table 1:** Patients undergoing pancreas transplantation.

Patients	*n*	Mean	Median
Age (range 29–66), years		46.8	47
Sex, female (%)	28(47.5)		
Race (%)		NA	NA
African American	20(33.9)		
Caucasian	35(59.3)		
Asian	1(1.7)		
Hispanic	3(5.1)		
Operation (%)		NA	NA
SPK	46(78.0)		
PAK	8(13.6)		
PTA	5(8.5)		
Type of pancreas Txp (%)		NA	NA
Primary	51(86.4)		
Second	6(10.2)		
Third	2(3.4)		
KDPI	NA	29	27
Cold Ischemic Time (CIT), hours	NA		
CIT Pancreas		10.1	7.9
CIT Kidney		11.2	10
Time (years) on HD before Txp	NA	1.32	0.48
Time (years) from listing to Txp	NA	0.7	0.29

**Table 2:** Graft failures and cause after pancreas transplantation.

Graft Survival (Days)	Cause of Failure
0	Thrombosis
93	Death
94	Rejection, pancreatectomy
30	Pseudoaneurysm
320	Rejection, no pancreatectomy

**Table 3:** Serum creatinine after pancreas transplantation.

Transplant Type	Creatinine	Follow up (Months, Mean)	Follow up (Months, Median)
PAK (n = 8)	1.37	6.1	4.9
PTA (n = 5)	1.01	4.0	4.1
SPK (n = 45)	1.24	7.4	6.8

PAK = pancreas after kidney transplant; PTA = pancreas transplant alone; SPK = simultaneous pancreas kidney transplant.

**Table 4:** Calcium scores and location (if available) for patients who underwent coronary artery assessment (CAA) with CT prior to pancreas transplantation.

Patient	Calcium Score	LMCA	LAD	Circumflex	RCA	LHC
1	0					No
2	0					No
3	0					No
4	0					No
5	0					No
6	0					No
7	0					No
8	0					No
9	0					No
10	7					No
11	22					No
12	24					No
13	28					Yes
14	71	0	21	12	37	No
15	79					No
16	136	67	69	0	0	No
17	142					No
18	170					Yes
19	184	0	132		3	Yes
20	214					Yes
21	287					Yes
22	1047	0	198	190	360	Yes
23	1060		889			Yes
24	5447	873	1836		2738	Yes

LMCA = left main coronary artery; LAD = left anterior descending artery; RCA = right coronary artery; LHC = left heart catheterization.

**Table 5:** Left heart catheterization (LHC) findings, location and percentage stenosis when data available. Note: Patients do not correlate to patients list in Table 4.

Patient	Calcium Score	LMCA	LAD Proximal	LAD Middle	LAD Distal	Diag	Circ	RCA	CABG
1		20%	10%					10%	
2		Normal	10–20%				100%	100%	
3	287	Normal	10–20%	30%		50%	30–40%	Normal	
4		Normal	20%				Normal	Normal	
5		Normal	20%					Normal	
6		Normal	20% ISS		40–50%		30%	20%	
7	5447	Normal	20%	30%			10%	20%	
8	1059	Calcification without stenosis	20%	20%	20%			Normal	
9		20–30%	20–30%						
10	1047	10%	10%	70%			25–40%	30%	Yes
11		Minor	40% ISS	40% ISS		40% ISS	Irregular	Irregular	
12		Normal	40–50%				Irregular	Normal	
13		Normal	40–60%			Normal	80%	Irregular	Yes
14	170	Normal	Minimal			20%	Normal	40%	
15			Minimal				Minimal	Minimal	
16	213.6	Normal	Moderate				Irregular	25%	
17		Normal	Mild				Normal	Minimal	
18		Normal		50%			None	None	
19	27.8	Normal		50%			Normal	Normal	
20				50%	60% ISS				
21		Normal	None	50%			90%	100%	Yes

LMCA = left main coronary artery; LAD = left anterior descending artery; Diag = diagonal artery; Circ = circumflex artery; RCA = right coronary artery; CABG = coronary artery bypass graft; ISS = in-stent stenosis.

**Table 6:** Comparison of CT scan only vs. LHC only for diagnosis of CAD in predicting troponin elevation after pancreas transplantation. Modality of assessment did not appear to predict troponin elevation (*p* = 0.20).

Troponin Elevation	CT Scan Only	LHC Only	Others
Troponin > 0.02	0	2	5
No elevation	17	9	26
Total	17	11	31

## Abbreviations

CAD	Coronary artery disease
CT	Computed tomography
CTA	Computed tomography with angiogram
DST	Dobutamine stress test
EF	Ejection Fraction
EST	Exercise Stress Test
KDPI	Kidney Donor Profile Index
LHC	left heart catheterization
MI	Myocardial Infarction
PAK	Pancreas after kidney transplant
PTA	Pancreas transplant alone
SPECT	Single photon emission computed tomography
SPK	Simultaneous pancreas-kidney transplant
